# NPreciSe - An Automated Satellite Precipitation Product Assessment Tool

**DOI:** 10.1038/s41597-024-03877-x

**Published:** 2024-09-27

**Authors:** Malarvizhi Arulraj, Veljko Petković, Susan Wen, Ralph R. Ferraro, Huan Meng

**Affiliations:** 1 Earth System Science Interdisciplinary Center/Cooperative Institute for Satellite Earth System Studies, University of Maryland, College Park, Maryland USA; 2 College of Computer, Mathematical, and Natural Sciences, University of Maryland, College Park, Maryland USA; 3 Center for Satellite Applications and Research, NESDIS/NOAA, College Park, Maryland USA

**Keywords:** Hydrology, Hydrology

## Abstract

Satellite-based Quantitative Precipitation Estimates (QPE) are indirect estimates of precipitation rates and as such are often prone to errors, warranting a need for characterizing the associated uncertainties before being used in application-specific studies. Moreover, multiple satellite-based QPE products are offered through different agencies, each with their own specifications, formats and requirements, posing a challenge to understanding the products uncertainties. This manuscript presents a standardized validation system named NPreciSe – NOAA Satellite-based Precipitation Validation System, which assesses the performance of satellite-based precipitation products in near real-time over the continental United States. NPreciSe is coupled with a user-interactive web platform and built using an open-source software, Python. It is structured to help (1) the end-users determine the best satellite QPE for their specific application, and (2) the algorithm developers identify systematic biases in QPE retrievals. This manuscript presents the capabilities of the NPreciSe, discusses the methodology adopted in developing the standardized validation system, and introduces the web portal.

## Introduction

With the advances in remote-sensing technologies and retrievals, most satellite-based QPEs now provide more than two decades of quasi-global precipitation data with high spatiotemporal resolution, thereby playing a major role in quantifying the global water cycle^1^, energy budget and water resources overall. Some satellite-based products derived from infrared-based sensors such as Precipitation Estimation from Remotely Sensed Information using Artificial Neural Networks-Climate Data Record (PERSIANN-CDR) provide QPEs going back to about four decades. The consistent and continuous QPE data record from satellite-based sensors serves as a primary input for weather forecasting, climate and hydrological modeling, data assimilation, natural hazard modeling, and risk assessments^2–7^. Moreover, satellite-based QPEs are the reliable estimates that can fill the gap in *in-situ* precipitation observations and radar precipitation estimates, especially over the ocean and remote areas^8^.

The US National Oceanic and Atmospheric Administration (NOAA), the National Aeronautics and Space Administration (NASA), and the Japan Aerospace and Exploration Agency (JAXA), along with their international partner agencies, support a plethora of satellite missions, with active and passive microwave (PMW) sensors to observe the precipitation systems globally with a high spatial and temporal resolution^9,10^. The satellite constellation consists of PMW sounders and imagers, including sensors such as the Special Sensor Microwave Imager/Sounder (SSMIS^11^), Microwave Humidity Sounder (MHS^12^), GPM Microwave Imager (GMI^13^), Advanced Microwave Scanning Radiometer 2 (AMSR2^14^) and Dual-frequency Precipitation Radar (DPR^15^) onboard different platforms. Furthermore, geostationary (GEO) satellites such as the Geostationary Operational Environmental Satellites-R (GOES-R^16^) series with imagers sensitive to visible and infrared wavelengths supplement the global monitoring of storms. Precipitation retrievals for individual sensors (Level-2) are designed based on specific sensor type, frequency channels, and targeted precipitation type following certain assumptions and methods. For example, both Goddard Profiling (GPROF^17,18^) and Microwave Integrated Retrieval System (MIRS^19–21^) are used to estimate instantaneous precipitation from PMW sensors but employ different methods. In their current versions, the former uses a fully parametric Bayesian inversion approach while the later inverts the observed radiances based on one-dimensional optimal estimation.

To support operational applications in different fields and develop consistent, user-ready precipitation records, instantaneous (Level-2) estimates from individual satellite sensors are often combined to derive the multi-satellite QPE products (Level-3). Examples include the Integrated Multi-satellitE Retrievals for GPM (IMERG^22^) and Climate Prediction Center morphing method (CMORPH^23^). Due to assumptions in retrievals and limitations in sensors’ sensitivity, the systematic and random errors associated with Level-2 QPEs propagate to the combined Level-3 products, compromising the application-specific studies^24^. Thus, given the multiple QPE sources and wide range of applications, understanding the pros and cons of each product is critical for enabling the users of diverse backgrounds to arrive at an informed decision.

The International Precipitation Working Group (IPWG) has been stressing the need for a systematic validation for the assessment of existing precipitation products since its formation in the early 2000s^25^. Several efforts have been conducted to organize high-quality ground observations worldwide to develop an operational and standardized validation system^26,27^. Kidd and Maggioni^28^ lists the past and current actions undertaken by the IPWG to build such a system for different regions with spatially dense ground-based observations. The present validation system provides a routine assessment of commonly used satellite precipitation products with a goal of helping users from diverse background to make an informed decision on the choice of precipitation product for their specific applications. For algorithm developers, the system provides near-real-time validation of their product and helps in the identification of any systematic or random retrieval errors. For national agencies responsible for delivering operational precipitation products for weather forecasting, the system facilitates standardized validation of in-house and other viable baseline products for consideration for potential upgrades and bridge the gap between research and operations.

To support agencies focusing on operational products, such as NOAA and aligning with the goal of the IPWG, a team of researchers and scientists from NOAA and partner research organizations outlined the guidelines and users’ preferences to develop a consistent, standardized, and operational validation system for satellite-based precipitation products^29^. The present work follows these guidelines through surveys and feedback from precipitation products developers and users to build an automated, standardized validation system for satellite-based precipitation products over the United States, named the NOAA Satellite Precipitation Validation System – NPreciSe. NPreciSe is a tool with an interactive user-oriented web portal that delivers free near-real-time assessments of Level-2 and Level-3 satellite precipitation products over the continental United States (CONUS). Specifically, the validation system evaluates both rainfall and snowfall rate estimates from Level-2 and QPEs from Level-3 products using high-quality ground observations from the Multi-Radar/Multi-Sensor (MRMS^30^) network as a reference. In addition to MRMS, NOAA National Centers for Environmental Prediction (NCEP) 4 km Stage IV precipitation data^31^ is also used as a reference for snowfall rate products. Moreover, NPreciSe facilitates the users to view the validation results based on a specific time or precipitation product. This article outlines the validation methodology, statistical metrics, capabilities, the tool itself, and future goals of NPreciSe.

## Results

The NPreciSe system consists of two modules: the validation module and the web portal module. The validation module assesses the detection and estimation performance of three types of satellite-based precipitation products such as Level-2 Rainfall Rate, Level-2 Snowfall Rate, and Level-3 accumulated precipitation using a standardized approach. The validation module produces an output comprising visualization and statistical metrics of the products’ performance within 1 to 12 hours of the satellite-based QPEs production. The web portal module displays the outputs produced by the validation module based on the queries requested by the users through an interactive webpage interface (https://precip-val.umd.edu/). This section describes the capabilities of the validation and web portal modules.

### Validation module

#### Level-2 instantaneous rainfall products

**Fig. 1:**
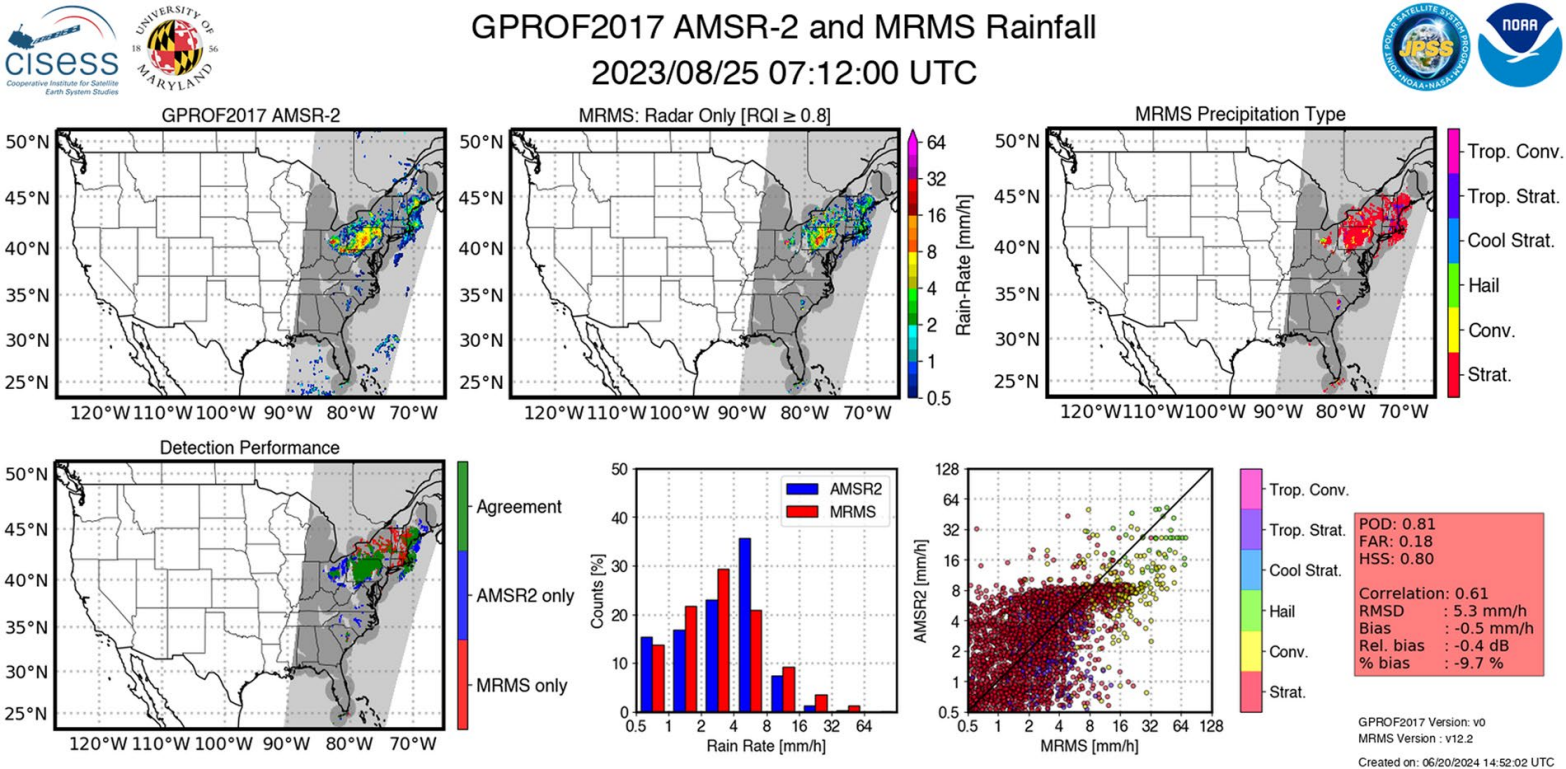
NPreciSe output for GPROF2017 GCOM-W1 AMSR2 product for August 25, 2023 precipitation event observed at 07:12 UTC. The template lists the satellite product name, reference product, and the corresponding overpass time in the header. The top row (from left to right) includes visualization of GPROF2017 QPEs, MRMS radar-only QPEs, and MRMS precipitation type estimates. The light gray shaded area depicts the satellite coverage, while the dark gray regions denote the availability of both satellite and reference products. The bottom row includes the spatial map showing the detection capabilities of satellites, the probability of distribution for the satellite and reference QPEs, a scatter plot, and the statistical metrics panel listing the detection and quantification performance. The template includes information on the version of the displayed satellite and reference products, along with the time stamp of the creation of the output file.

The validation output of Level-2 rainfall products includes visualization of the precipitation estimated by the satellite-based sensor and MRMS radars. A map of precipitation type information from the MRMS network is included to provide information on the precipitating storm regime to the users. The detection capabilities of the retrieval are depicted through a spatial map, and the corresponding statistical metrics. For all the footprints where both the satellite and reference product detected precipitation accurately, a probability distribution function (PDF), a scatter plot, and statistical metrics are calculated to highlight the quantification performance of the satellite product. The scatter plots are color-coded by the precipitation type to provide additional information on the satellite-based QPE’s performance under different precipitation conditions. Figure 1 shows an example validation output of the Level-2 satellite-based rain rate products. This example evaluates the GPROF retrievals from the AMSR2 sensor on board GCOM-W1 with corresponding MRMS estimations for the August 25, 2023, precipitation event. The MRMS precipitation type data suggest mostly stratiform precipitation with some hail and convective activities. The detection map in the bottom panel shows that AMSR2 captures most of the storm’s spatial structure well, but there are some misses and false alarms. The MRMS PDF depicts heavy tails compared to the AMSR2, suggesting an underestimation of heavy precipitation. The scatter plot further confirms that the underestimated heavy precipitation is predominantly from convective and hail precipitation types. The red box lists the detection and quantification metrics providing an overall statistical score for the performance of the satellite product.

#### Level-2 instantaneous snowfall rate products

**Fig. 2:**
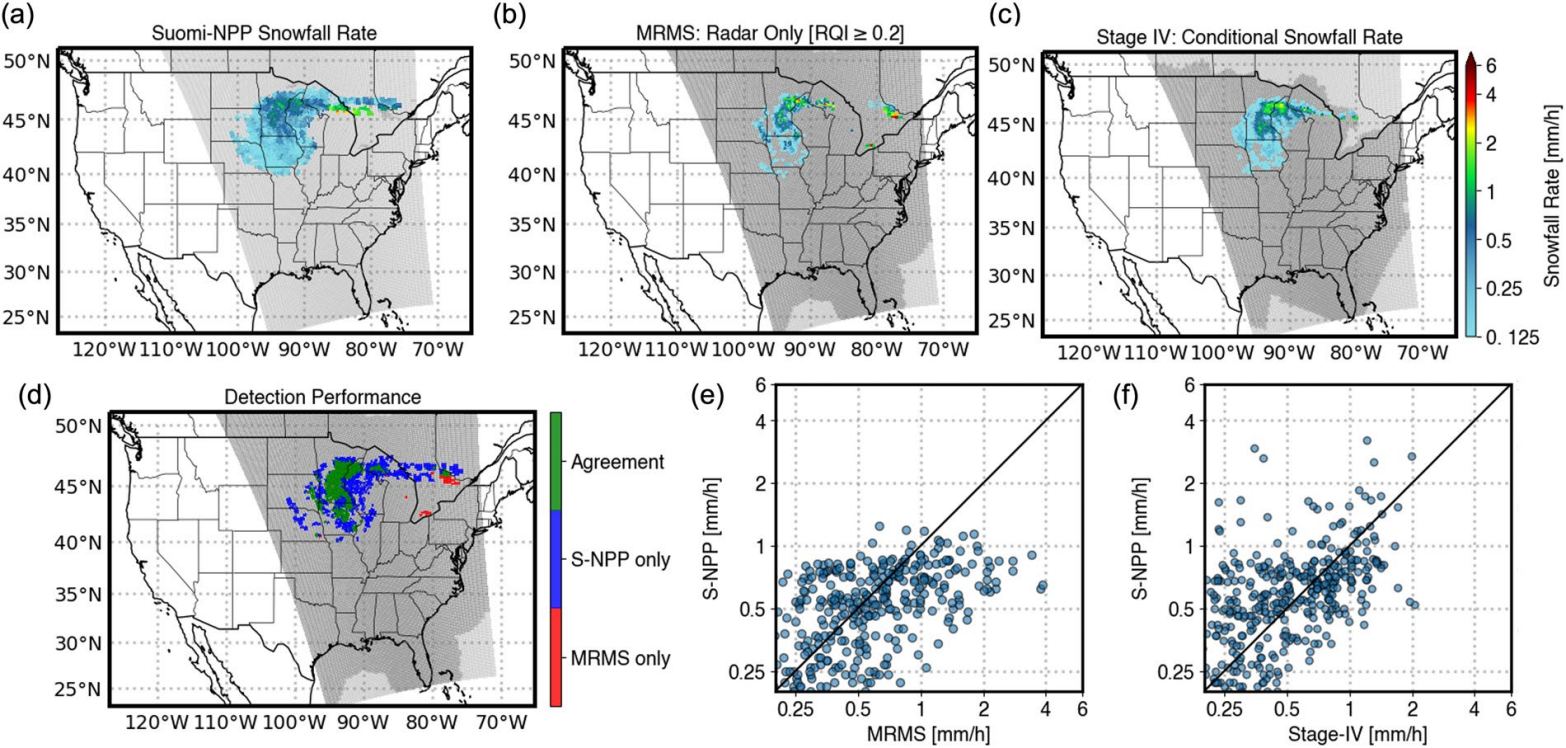
Validation of Suomi-NPP SFR product using MRMS and Stage IV snowfall rate estimates. (**a**) SFR estimates by Suomi-NPP SFR product, (**b**) Snowfall rate observed by MRMS, and (**c**) Precipitation Rate by Stage IV conditional on Suomi-NPP SFR estimates for January 4, 2023, at 18:42 UTC. (**d**) The detection performance of Suomi-NPP SFR when compared to MRMS; Scatterplots comparing snowfall estimates from Suomi-NPP with (**e**) MRMS and (**f**) Stage IV estimates. Note the lighter gray shades denote satellite overpass, and the darker shaded regions denote MRMS observations with RQI > 0.2 and Stage-IV coverage region.

Level-2 Snowfall Rate (SFR) products are validated using MRMS and Stage-IV precipitation products. The template for Level-2 SFR validation with MRMS as a ground reference is very similar to that of the Level-2 rain-rate template, with two notable differences. The MRMS precipitation type panel in Fig. 1 will not be available in the Level-2 SFR validation since only precipitation estimates of snow are considered for validation. For assessment with Stage-IV as a reference, since the instantaneous Level-2 SFR products are compared with 1-hour accumulated Stage-IV data that do not include precipitation type information, the output template will not have the spatial map of precipitation type, detection performance, and detection metrics. The ability of NPreciSe to handle SFR products and their assessment against different reference products is demonstrated in Fig. 2. In this example case the North-Central US snowfall event, occurred on January 4, 2023, is captured by the Suomi-NPP ATMS overpass. Figure 2(a–c) shows the spatial map of the snowfall rate delivered by the ATMS sensor, Stage-IV, and MRMS, respectively. Since Stage-IV products are hourly-accumulated and do not include precipitation type and phase, the map in Fig. 2(c) shows Stage IV precipitation estimates over the areas where Suomi-NPP ATMS detects snowfall. Figure 2(d) shows the detection performance of Suomi-NPP ATMS against MRMS product. Note that the MRMS SFR estimates miss light snowfall captured by the Suomi-NPP in this case example. A similar map for Stage IV as a reference product cannot be calculated due to the absence of precipitation phase information. The scatter plots highlight that the correctly detected events are relatively close to the 1-to-1 line for the Stage-IV and MRMS products for this particular overpass of Suomi-NPP. The statistical metrics for this event are shown in Table 1. Note that the detection metrics for Stage-IV as reference is not applicable.

**Table 1:** Validation metrics for the Suomi-NPP SFR estimates for the January 4, 2023 case shown in Fig. 2.

Evaluation Metric [units]	Reference Product	
	MRMS	Stage IV
Probability of Detection (POD)	0.95	—
False Alarm Ratio (FAR)	0.58	—
Heidke Skill Score (HSS)	0.56	—
Correlation Coefficient (CC)	0.49	0.48
Root Mean Square Deviation (RMSD) [mm h^−1^]	0.5	0.4
Absolute Bias (AB) [mm h^−1^]	−0.0	0.1
Percent Bias (PB) [%]	−7.9	34.5
Relative Bias (RB) [dB]	−0.4	1.3

#### Level-3 precipitation products

**Fig. 3:**
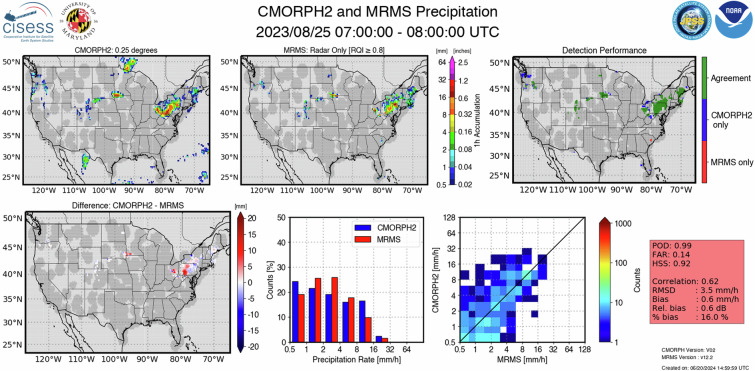
NPreciSe output of hourly-accumulated CMORPH2 QPEs validated with MRMS precipitation estimates for August 25, 2023. The top panel shows the spatial map of CMORPH2 QPE, MRMS precipitation estimates, and the detection performance of CMORPH2. The bottom panel shows the difference, PDFs, and a scatter plot between the CMORPH2 and MRMS estimates, along with the detection and quantification metrics. The light gray shades in the spatial map denote the coverage of the satellite-based product only. The dark gray shades denote the availability of good-quality reference and satellite products.

The validation output for accumulated precipitation products includes spatial maps of satellite-based and reference QPE, the satellite product detection and quantification performance, the PDFs of precipitation estimates, and a density-based scatter plot. Figure 3 shows an example validation output of hourly-accumulated Level-3 precipitation products for CMORPH2 validated against 1-hour MRMS precipitation. Level-3 products with higher than 10-minute temporal resolution follow the same output format as in Fig. 1 as the reference in that case is the 2-minute MRMS product.

### Web portal module

**Fig. 4:**
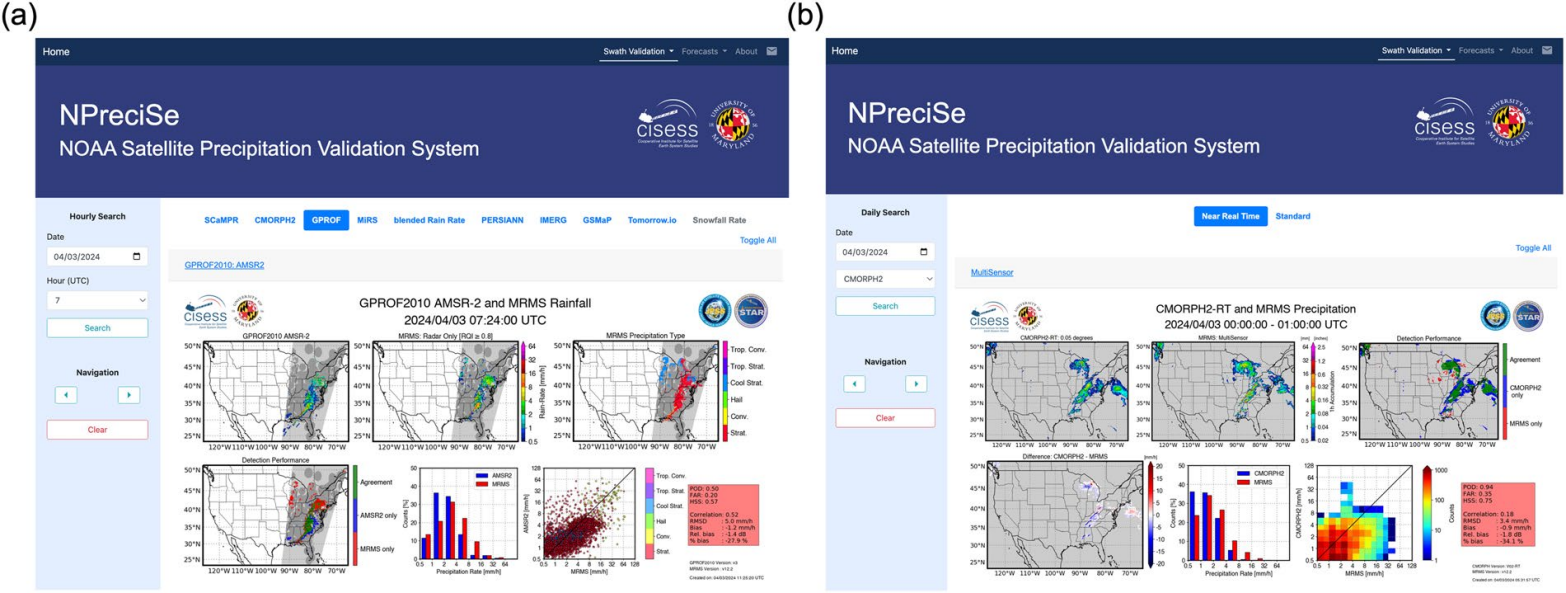
NPreciSe web portal (https://precip-val.umd.edu/). Output of user-interactive platform: (**a**) Validation by Time, and (**b**) Validation by Product option.

The web portal module is the frontend of the NPreciSe package that facilitates users to access the real-time and archived assessment of satellite-based precipitation products. The NPreciSe page can be accessed through a secured URL: https://precip-val.umd.edu/. Two options are offered to the users on the homepage of NPreciSe to access the output of the validation modules, namely, “Validation by Time” and “Validation by Product”. The “Validation by Time” option enables the users to view the assessment of all the precipitation products for a particular day. This option benefits users interested in deciding the best satellite-based product for a specific precipitation event or storm. An illustration of this query is shown in Fig. 4(a). The user inputs the date, and time (in hours) through the search panel in the webpage’s sidebar. As the query is submitted, NPreciSe displays the validation outputs of all the satellite-based products of the specified precipitation type for the chosen date and time. The second option, “Validation by Product”, is for users interested in a particular product or algorithm, where the user has an option to search the assessment of a particular satellite-based product for any particular day/hour. Under this option, when the user submits the date and precipitation product name, NPreciSe presents the corresponding validation outputs. Figure 4(b) shows an illustration of the “Validation by Product” query. If the validation output of a particular product is not available at the chosen time, a message is displayed to notify the user.

## Discussion

With a wide range of satellite-based precipitation estimates available through various international agencies, and with end-application users of diverse backgrounds, choosing a product that best suits the needs of specific application is challenging. Besides, the products often follow different file specifications, formats, and variable names, being distributed to the public through various portals such as HTTPS, FTP, or customized web application-based systems. Even when those portals distribute the assessment of the products, the methodology followed and statistics included vary significantly between different products, thus further challenging the intercomparison. For users of precipitation products, a standardized system such as NPreciSe provides a comprehensive evaluation of satellite-based precipitation estimates, enabling application-specific informed decisions. NPreciSe will serve both the users and developers as a one-stop resource offering its routine validation in support to national agencies, such as NOAA, in decision-making, planning and upgrading of current and next generation products.

**Fig. 5:**
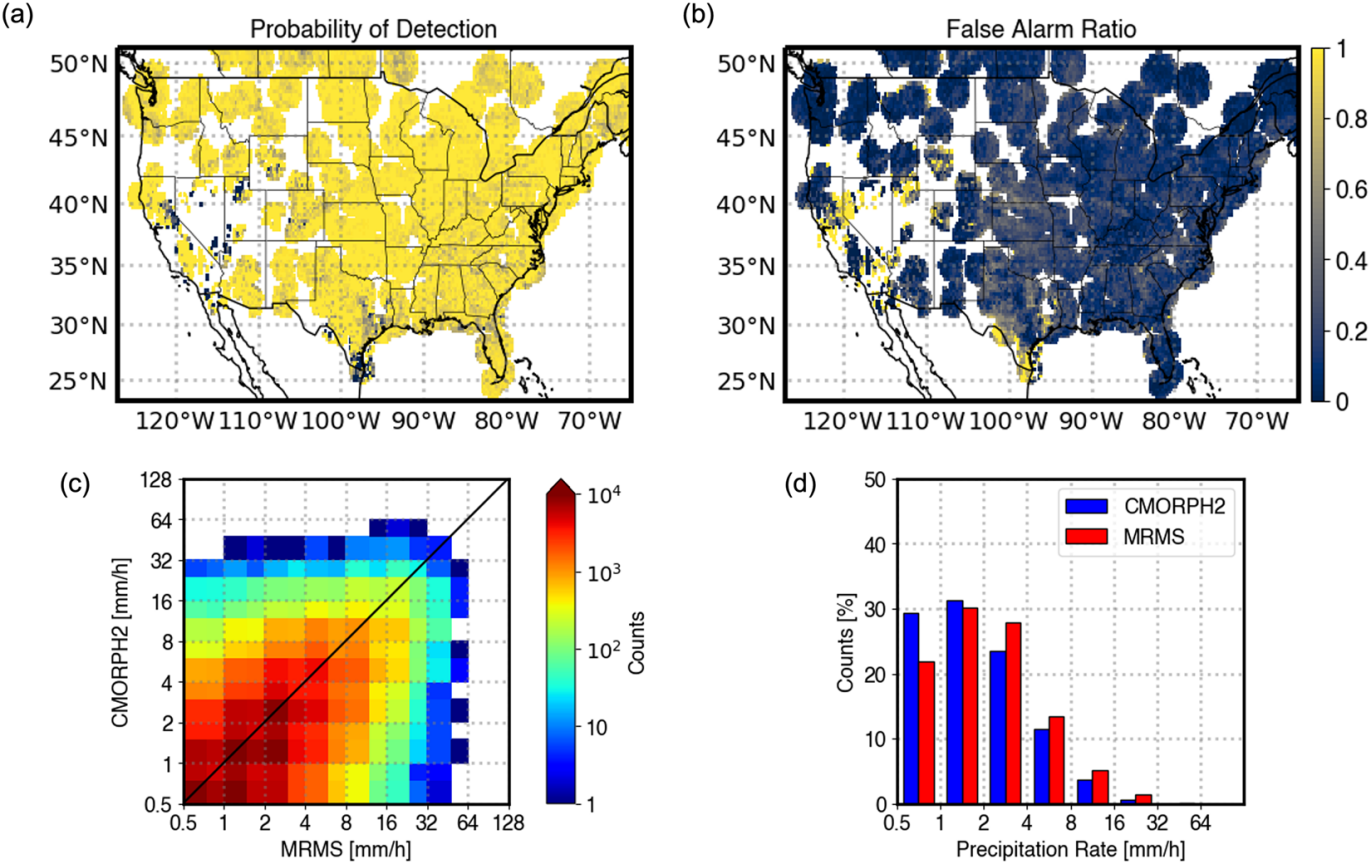
Monthly validation of CMORPH2 for June 2022. Spatial maps of (**a**) Probability of Detection and (**b**) False Alarm Ratio. (**c**) Density-based scatter plot and (**d**) Probability Distribution Function of CMORPH2 using hourly-accumulated MRMS RadarOnly Product as a reference.

NPreciSe currently includes the commonly used and publicly available satellite-based precipitation datasets. However, the list of precipitation products will continue to expand to accommodate new datasets, and a new version of the current products when released. Both the validation and web application module of NPreciSe is developed using open-source software and packages such as Python, Cartopy and FlaskApp, enabling transferability of the system to include different products and regions straight-forward. Moreover, in addition to the individual swath-based validation currently included in the system, the instantaneous matchups produced by NPreciSe can be used to calculate monthly or annual validation statistics. An example of monthly validation for CMORPH2 is shown in Fig. 5. During this month, most regions of the CONUS exhibit high POD and low FAR, suggesting high detection skill scores. CMORPH2 false alarms were dominant in the western US and southern part of Texas. The density scatterplot indicates high correlation between the CMORPH2 and MRMS products with the correlation coefficient of 0.46 and bias of −0.5 mm/h. When analyzing the percent bias with respect to precipitation rate, a typical behavior of satellite products is noted – overestimation of light precipitation and underestimation high precipitation rates. More detailed analysis, such as the detection and quantification scores by precipitation type, over specific region, or season, can also be performed using the data created within the NPreciSe package.

## Methods

This section describes the precipitation products included in NPreciSe, the reference products considered, and methodology followed in the validation module.

### Data description

#### Satellite-based precipitation products in NPreciSe

NPreciSe validates three types of satellite-based precipitation products: Level-2 Rainfall Rate, Level-2 Snowfall Rate, and Level-3 accumulated precipitation. The instantaneous Level-2 products validated in NPreciSe include GPROF^17,18^ and MIRS^19–21^ rainfall rate products and snowfall rate retrieval (SFR^32,33^) for sensors such as AMSR2, ATMS, SSMIS, MHS, and GMI. In addition to the individual sensors, NPreciSe validates rainfall estimates from NOAA’s Blended Hydro product. Blended rain-rate (bRR^34^) product offers a unified QPE field, primarily for weather forecasters, by blending the QPEs from multiple PMW sensors. bRR combines the instantaneous precipitation estimates within 10-hour time-period from multi-satellites to generate a gridded output and is commonly used by the National Weather Service forecasters. The sampling resolution of the Level-2 products is considered to be instantaneous, matching the field of view (FOV) of the observing sensors (5 km to 60 km) which typically take 5–10 minutes to complete an overpass of the CONUS. Each of the products are ingested into NPreciSe at a different point of time. Table 2 lists the Level-2 rainfall and snowfall rate products evaluated in NPreciSe, the minimum detectable threshold and the date of availability of validation results.

**Table 2:** Level-2 Rainfall and Snowfall Rate Products evaluated in NPreciSe.

Retrieval	Sensor	Satellite	Availability	Minimum detection threshold [mm/h]	Reference Product
GPROF	AMSR-2	GCOM-W1	November 6, 2020 - present	0.5	MRMS Radar estimates [Temporal resolution = 2 minutes; RQI > = 0.8; Precipitation type other than snow]
MIRS	ATMS	NOAA-20	December 8, 2020 - present	0.5	
		NOAA-21	August 20, 2023 - present		
		Suomi-NPP	December 12, 2020 - present		
	SSMIS	DMSP F17	February 9, 2022 - present	0.5	
		DMSP F18	February 9, 2022 - present		
	MHS	NOAA-19	February 9, 2022 - present	0.5	
		MetOp-B	February 9, 2022 - present		
		MetOp-C	July 10, 2023 - present		
	GMI	GPM	February 9, 2022 - present	0.5	
bRR	Multi satellite		November 3, 2020 - present	0.5	
SFR	ATMS	NOAA-20	April 16, 2021 - present	0.125	1) MRMS Radar estimates [Temporal resolution = 2 minutes; RQI > = 0.2; Precipitation type = snow] 2) Stage-IV precipitation estimates [Temporal resolution = 1 hour]
		Suomi-NPP	April 16, 2021 - present		
	SSMIS	DMSP F17	January 3, 2022 - present	0.125	
		DMSP F18	January 3, 2022 - present		
	MHS	NOAA-19	January 3, 2022 - present	0.125	
		MetOp-B	January 3, 2022 - present		
		MetOp-C	January 3, 2022 - present		
	GMI	GPM	January 3, 2022 - present	0.125	

Level-3 multi-sensor precipitation products assessed in NPreciSe currently include: CMORPH2^23,35^, SCaMPR^36,37^, IMERG^22,38^, GSMaP^39,40^, and PERSIANN^41–43^. The Level-3 products deliver gridded spatially continuous QPEs relying on low Earth orbiting satellite observations and, more often, geostationary sensors operating at infrared and visible frequencies. Most Level-3 products also incorporates information from other high-quality satellite-based estimates (Level-2 PMW-based QPEs) and ground-based observations. Table 3 lists the sources and resolution of the Level-3 products assessed in NPreciSe. Contrary to the Level-2 products, Level-3 products deliver precipitation estimates that include rain and snow and have a temporal resolution between 10 and 60 minutes. Similar to Level-2 products, each of the Level-3 precipitation products are added to NPreciSe at a different point of time. Table 4 summarizes the Level-3 precipitation products included in NPreciSe, time-period at which validation results are available and the minimum detection threshold.

**Table 3:** List of Level-3 products considered in NPreciSe.

Product	Source	Latency [hours]	Spatial Resolution [degrees]	Temporal Resolution [minutes]
CMORPH2 Near Real Time	IR coupled with PMW observations and model precipitation forecasts from NCEP operational global forecast system	5	0.05	30
CMORPH2 Standard	Same as CMORPH2 Near Real Time – but with more predictors	36	0.25	
SCaMPR	IR at 6, 7, 8.5, 11 and 12 μm calibrated against the CPC combined microwave (MWCOMB) dataset	<1	0.02	10
IMERG-Early	Forward propagation of IR at 11 μm, PMW estimates, and bias corrected with gauges	4	0.10	30
IMERG-Late	Same as IMERG-Early; forward and backward propagation	14		
GSMaP-NRT	IR combined with PMW observations and bias corrected with gauges	4	0.10	60
GSMaP-MVK	Same as GSMaP-NRT – but with more predictors	72		
PERSIANN DIR-Now	IR at 11 μm; trained and bias adjusted with IMERG merged PMW observations	<1	0.04	60
PERSIANN	IR at 11 μm; trained with MWCOMB dataset	48	0.25	60

**Table 4:** Description of Level-3 Precipitation Products evaluated in NPreciSe.

Product	Availability	Minimum Detection Threshold [mm/h]	Reference Product
CMORPH2 Near Real Time	May 11, 2022 - present	0.5	1) MRMS Radar Only precipitation [Temporal resolution = 1 hour; RQI > = 0.8] 2) MRMS MultiSensor precipitation [Temporal resolution = 1 hour]
CMORPH2 Standard	May 5, 2022 - present	0.5	
SCaMPR	March 1, 2021 - present	1.0	MRMS Radar estimates [Temporal resolution = 2 minutes; RQI > = 0.8]
IMERG-Early	December 28, 2021 - present	0.5	1) MRMS Radar Only precipitation [Temporal resolution = 1 hour; RQI > = 0.8] 2) MRMS MultiSensor precipitation [Temporal resolution = 1 hour]
IMERG-Late	December 28, 2021 - present	0.5	
GSMaP-NRT	July 25, 2022 - present	0.5	
GSMaP-MVK	April 1, 2022 - present	0.5	
PERSIANN DIR-Now	October 12, 2021 - present	0.5	
PERSIANN	November 14, 2021 - present	0.5	

#### Reference products in NPreciSe

The MRMS network provides three-dimensional mosaics of precipitation estimates over the CONUS by ingesting observations from 180 S-Band dual-polarization “Weather Surveillance Radar-1988 Doppler” (WSR-88D) radars^30^. The data are operationally distributed to the public in real time through NOAA NCEP platform. MRMS provides precipitation data with a spatial resolution of 0.01 degrees and a temporal resolution of as low as 2 minutes. In addition to the precipitation products derived solely from radars (“RadarOnly”), MRMS also includes accumulated precipitation estimates from combined products namely, the MultiSensor product. The MultiSensor product is derived from the radars, 7,000 gauges, and Rapid Refresh numerical weather prediction model output^44^ and has a minimum temporal resolution of one hour. The 2-minute temporal resolution product includes three variables, namely, “PrecipRate”, “PrecipFlag” and “RadarQualityIndex”. The “PrecipRate” provides the precipitation rate from radars in mm/h, while the “PrecipFlag” delivers corresponding type of the precipitation. The precipitation types include warm stratiform rain, snow, convective, hail, tropical/stratiform mix, tropical/convective mix, and cool stratiform rain^45^. The “RadarQualityIndex” (RQI) parameter comprises the quality of observations, ranging from 0 to 1, with 1 denoting the highest quality data. Moreover, hourly accumulated precipitation estimates from “MultiSensor” and “RadarOnly” products are also used in NPreciSe as reference data. The spatial coverage of the “RadarOnly” product varies based on the availability of radar observations, and beam blockage while the MultiSensor product comprises seamless QPEs over CONUS and does not include a quality flag. Note that MRMS produces high-quality liquid precipitation observations. However, snowfall events with less than 5 mm daily accumulation are severely underestimated^46^.

The NCEP Stage IV data include multi-sensor regional analysis of precipitation produced by the twelve River Forecast Centers (RFCs) over the CONUS, with a spatial resolution of approximately 4 km and hourly sampling^31^. Stage IV estimates primarily rely on observations from ~140 weather radars and are bias corrected using measurements from 5,500 rain gauges. While the Stage IV data are widely used as a ground reference product for validating satellite-based precipitation, including the snowfall rate^47–50^, caution is warranted over the regions of the western United States and outside the RFCs^51^. NPreciSe uses Stage IV data as a reference for snowfall rate products only.

### Validation module

To validate satellite-based precipitation products, NPreciSe adapts a standardized approach in a three-step sequence: 1) Identifying the best reference, 2) Collocating of satellite and reference products, and 3) Calculating statistical metrics to quantify the detection and estimation performance of satellite-based products. While the method is uniformly applied across all products, minor changes are followed to accommodate satellite-based QPEs based on the precipitation type and spatial/temporal resolution. The methods followed for different categories of satellite-based precipitation products and the adapted evaluation metrics are explained in this sub-section.

#### Identification and collocation of reference products

**Fig. 6:**
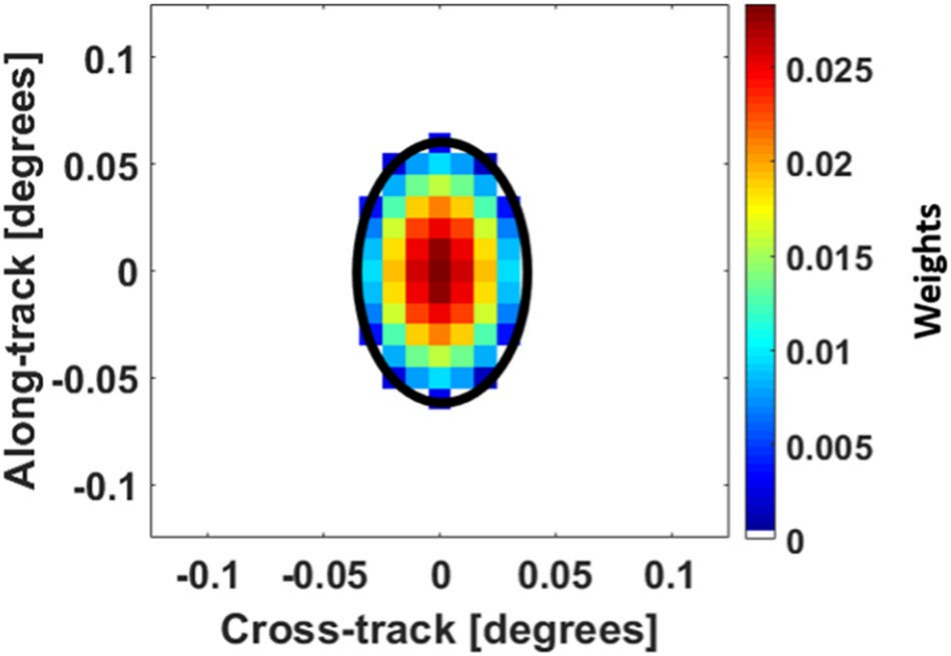
Representation of Level-2 satellite-based (black ellipse) and MRMS (squares) field of view. Colors denote standardized weights applied to the MRMS product within the AMSR2 36-GHz channel footprint.

Level-2 Rainfall Products: With their temporal resolution of 2 minutes, the MRMS radar only products are best suited to serve as a reference in evaluating instantaneous satellite-based products (Level-2; Table 2). To maintain the best quality and minimize the uncertainty of the MRMS product, only observations with a radar quality index greater than or equal to 0.8 are considered for constructing the reference data. The value 0.8 is chosen based on MRMS developers’ recommendations. Moreover, pixels identified as snow by MRMS are not considered for validating the instantaneous rain-rate products. Even though MRMS radar only products have gaps due to coverage, MultiSensor product is not used for the validation of Level-2 products due to its coarser temporal resolution (1 hour). After choosing the reference product, the next step in validation process is matching the collocation of the Level-2 products and MRMS observations. The Level-2 products provide instantaneous scans, with each adjacent scan differing only by a few seconds. Most PMW sensors overpass the CONUS region within approximately 5–10 minutes. NPreciSe utilizes the mid-point of time between the start and end of the CONUS overpass to match with the nearest 2-minute MRMS observations. Following this strategy, the instantaneous time scans by the PMW sensors are compared with the ground observations made within 5–7 minutes of the overpass. The Level-2 rain-rate products follow the spatial resolution of the corresponding PMW sensor’s footprint. MRMS QPE estimates are of finer spatial resolution than any Level-2 rain-rate products. To allow for one-to-one comparison, the two are collocated by calculating a distance-weighted mean of MRMS grids within the satellite footprints. The weights for the MRMS grids are calculated following an idealized antenna pattern (i.e., 2D Gaussian), with the MRMS grids closer to the center of the footprint receiving the highest weight. The spatial depiction of the satellite footprint, MRMS grids, and their associated weights is shown in Fig. 6.

Level-2 Snowfall Rate Products: Level-2 SFR products have the same temporal and spatial resolution to the Level-2 rainfall rate products. Even though 2-minute MRMS products would be expected to provide an ideal reference for the validation of snowfall rate products, the challenges of ground radar algorithm result in limited ability to accurately retrieve snow regimes, especially light snowfall^46^. Thus, NPreciSe considers snowfall rate estimates from both the 2-minute MRMS and hourly-accumulated Stage-IV precipitation data as the reference product. When using MRMS as a reference product, an RQI value of 0.2 or greater is considered to be a good quality observation, based on the guidance provided by the MRMS science team. On the other hand, a temporal resolution of 1 hour and a lack of precipitation type estimate in Stage-IV products presents its own challenges to the validation process. Therefore, the Stage-IV product is not used to assess the detection capabilities but only to characterize the performance of satellite-based SFR products. You *et al*.^52^ reports a time lag of approximately 30 minutes between the satellite-based PMW estimates of surface snowfall and the ground observations. The study highlights that the lag is due to the slower fall speed of snow (compared to liquid hydrometeors). Thus, the instantaneous SFR products are evaluated against 30-minute lagged 2-minute MRMS observations to account for the snow-falling speed. Since the Stage-IV precipitation estimates are hourly accumulations, and keeping the 30-minute lag in mind, the nearest hourly Stage-IV file to the SFR overpass time is considered the reference. When performing the spatial matching of SFR and the reference product’s footprint, the same approach to that of the instantaneous rain-rate products is followed (Fig. 6).

Level-3 Precipitation Products: Level-3 precipitation products have various temporal resolutions ranging from 10 minutes to 1 hour. If more frequent than hourly and less frequent than 10 minutes, NPreciSe aggregates Level-3 precipitation products to the hourly time scale, assuming a constant precipitation rate between available observations, where applicable. On the other hand, the same timescale is retained for precipitation products with 10-minute temporal resolution, such as SCaMPR (see Table 3). The reference data for 1-hour precipitation accumulation includes MRMS’s “RadarOnly” and “MultiSensor” products, while the 10-minute satellite-based estimates are assessed against 2-minute MRMS radar observations. Note that the “RadarOnly” products are developed using precipitation estimates from radar observations, while the “MultiSensor” product combines radar data with gauges and numerical weather prediction forecasts, providing a holistic evaluation over the regions lacking radar coverage. Similar to Level-2 product evaluation, an RQI of 0.8 is considered as a threshold for MRMS “RadarOnly” 2-minute products. Finally, to match the spatial resolution of Level-3 products and ground reference, MRMS fine-scale reference product grids (0.01°) are averaged over the corresponding product’s spatial resolution. Note, NPreciSe retains the native spatial resolution of the satellite precipitation products.

#### Calculation of statistical metrics

After matching the spatial resolution of the satellite-based and ground-based products, the system calculates the statistical metrics to quantitatively represent the detection and estimation capabilities of the satellite-based precipitation products. The detection metrics highlight the satellite products’ ability to represent precipitation extent accurately. The minimum sensitivity of the satellite-based precipitation products usually determines the threshold for detecting precipitation. The thresholds for detecting precipitation are set to values ranging from 0.125 mm/h to 1 mm/h, matching the product’s minimum detection capability. The detection threshold of all the Level-2 and Level-3 precipitation products are listed in Table 2 and Table 4. The detection thresholds of Level-2 SFR products and rainfall products are 0.125 mm/h and 0.5 mm/h, respectively. All the Level-3 products except SCaMPR have a threshold of 0.5 mm/h while the detection threshold for SCaMPR is 1.0 mm/h. These thresholds are used to determine the correct detections (CD), false alarms (FA), missed detection (MD), and correct misses (NN). CDs are identified when the satellite and reference products report precipitation rates above the threshold. FA denotes the instance where satellite detects precipitation while the reference records no precipitation. When the reference product observes precipitation above the threshold, but the satellite fails to detect precipitation, the event is termed MD. Finally, the NN suggests that the satellite and reference products detect no precipitation. Some of the commonly used detection metrics are the Probability of Detection (POD^53^), False Alarm Ratio (FAR^53^) and Heidke Skill Score (HSS^54^). For the CD instances, the quantification performance of the satellite-based sensors is computed by the following metrics: Correlation Coefficient (CC), Root Mean Square Deviation (RMSD), Absolute Bias (AB), Percent Bias (PB) and Relative Bias (RB^55,56^). The positive values of AB, PB, and RB indicate overestimation of precipitation, while the negative values denote underestimation. The mathematical formulation of the metrics and the ideal scores are shown in Table 5.

**Table 5:** Statistical Metrics considered in NPreciSe to quantitatively assess the detection and quantification capabilities of precipitation products.

Statistical Metrics	Category	Definition	Score Range (Ideal Score)
Probability of Detection (POD)	Detection	$\frac{{CD}}{{CD}+{MD}}$	[0, 1] (1)
False Alarm Ratio (FAR)	Detection	$\frac{{FA}}{{CD}+{FA}}$	[0, 1] (0)
Heidke Skill Score (HSS)	Detection	$\frac{2\times (\left({CD}\times {NN}\right)-\left({FA}\times {MD}\right))}{\left({CD}+{MD}\right)\left({MD}+{NN}\right)+({CD}+{FA})({FA}+{NN})}$	[0, 1] (1)
Correlation Coefficient (CC)	Estimation	$\frac{\sum ({R}_{sat}\mbox{--}\,\overline{\,{R}_{sat}}\,)({R}_{ref}\mbox{--}\,\overline{\,{R}_{ref}}\,)}{\sqrt{\sum {({R}_{sat}\mbox{--}\overline{{R}_{sat}})}^{2}\sum {({R}_{ref}\mbox{--}\overline{{R}_{ref}})}^{2}}}$	[−1, 1] (1)
Root Mean Square Deviation (RMSD)	Estimation	$\sqrt{\sum \frac{{({R}_{sat}-{R}_{ref})}^{2}}{N}}$	[0, inf] (0)
Absolute Bias (AB)	Estimation	$\frac{1}{N}(\sum {R}_{sat}-\sum {R}_{ref})$	[-inf,inf] (0)
Relative Bias (RB)	Estimation	$10log\left(\frac{\sum {R}_{sat}}{\sum {R}_{ref}}\right)$	[-inf, inf] (0)
Percent Bias (PB)	Estimation	$\frac{\sum {R}_{sat}-\sum {R}_{ref}}{\sum {R}_{ref}}\times 100$	[-inf, inf] (0)

CD, FA, MD and NN denote correct detections, false alarms, missed detections and correct misses, respectively. *R*_*sat*_ and *R*_*ref*_ denote the precipitation estimates from satellite-based and reference products while $\bar{\,{R}_{{sat}}}$ and $\bar{\,{R}_{{ref}}}$ denote the mean values of satellite-based and reference products.

## Data Availability

The assessment of satellite precipitation products is available for download through online web portal: https://precip-val.umd.edu. The web portal is updated in real-time.

## Code availability

A complete package containing Python scripts, installation, and processing instructions for matching the footprints of satellite products and generating the plots presented throughout the manuscript (as well as those available on the NPreciSe portal) is available at https://code.umd.edu/marulraj/nprecise.git.
